# Japanese encephalitis virus infects porcine kidney epithelial PK15 cells via clathrin- and cholesterol-dependent endocytosis

**DOI:** 10.1186/1743-422X-10-258

**Published:** 2013-08-12

**Authors:** Songbai Yang, Minhui He, Xiangdong Liu, Xinyun Li, Bin Fan, Shuhong Zhao

**Affiliations:** 1 Key Laboratory of Agricultural Animal Genetics, Breeding and Reproduction, Ministry of Education & College of Animal Science and Technology, Huazhong Agricultural University, Wuhan 430070, China

**Keywords:** JEV, PK15, Cholesterol, Caveolin-1, Clathrin, Infection

## Abstract

**Background:**

Japanese encephalitis virus (JEV) is a mosquito-borne flavivirus that causes acute viral encephalitis in humans. Pigs are important amplifiers of JEV. The entry mechanism of JEV into porcine cells remains largely unknown. In this study, we present a study of the internalization mechanism of JEV in porcine kidney epithelial PK15 cells.

**Results:**

We demonstrated that the disruption of the lipid raft by cholesterol depletion with methyl-β-cyclodextrin (MβCD) reduced JEV infection. We also found that the knockdown of clathrin by small interfering RNA (siRNA) significantly reduced JEV-infected cells and JEV E-glycoprotein levels, suggesting that JEV utilizes clathrin-dependent endocytosis. In contrast, the knockdown of caveolin-1, a principal component of caveolae, had only a small (although statistically significant) effect on JEV infection, however, JEV entry was not affected by genistein. These results suggested that JEV entry was independent of caveolae.

**Conclusions:**

Taken together, our results demonstrate that JEV enters porcine kidney epithelial PK15 cells through cholesterol- and clathrin-mediated endocytosis.

## Background

Japanese encephalitis virus (JEV) is a mosquito-borne flavivirus that belongs to the family Flaviviridae. JEV is one of the most important endemic encephalitides and can cause acute viral encephalitis, of which there are approximately 50,000 cases in humans annually [1]. JEV can infect a wide range of cells of different origins. Pigs act as amplifying hosts of JEV; therefore, the domestic pig was considered to be a risk factor in the transmission of the disease to humans [2,3]. JEV is also an important pathogen in swine and causes considerable economic losses in pork production. The primary symptoms of pigs infected with JEV are fetal abortion and stillbirth in infected sows and aspermia in boars [4,5]. JEV has a single-stranded positive-sense RNA genome of approximately 11 kb. The viral RNA encodes a single large polyprotein that is cleaved into three structural proteins, capsid (C), precursor membrane (prM) and envelope (E); and seven non-structural (NS) proteins, NS1, NS2a, NS2b, NS3, NS4a, NS4b and NS5. The JEV E protein is the major structural protein exposed on the surface of the virus particle and mediates binding and fusion during virus entry [6,7].

Viruses enter cells through binding cellular receptors. The interactions between the viruses and receptors are highly specific, determining which cell types and species can be infected. Additionally, the entrance of viruses into the host cells involves several endocytic pathways, including clathrin-mediated, caveolae-mediated, cholesterol-dependent endocytosis, macropinocytosis/phagocytosis and other mechanisms [8,9]. Clathrin-mediated endocytosis (CME) is the best characterized of the endocytic mechanisms, and most viruses utilize this type of endocytosis to enter cells. Recent studies have shown that JEV infects neuronal cells through a clathrin-independent, dynamin- and caveolae-mediated endocytosis pathway [10,11]. Previous studies have found that JEV enters Vero and Huh7 cells through a clathrin-dependent pathway [12,13]. In addition, JEV internalisation into neural stem cells occurs by clathrin-mediated, caveolae independent endocytosis [14]. Persistent JEV infection has been demonstrated in porcine kidney cells [15] and numerous studies on JEV have been conducted in porcine kidney cells [16-20]. Moreover, vimentin has been identified as mediating the entry of JEV into porcine kidney cells [21]. However, the precise entry mechanism for JEV internalization into porcine cells remains unclear.

In this study, we define the role of cholesterol in JEV infection through cholesterol depletion, which significantly decreased JEV infection. In addition, we used RNA interference (RNAi) to examine the roles of clathrin and caveolin-1 in the JEV entry process; the results indicated that knockdown of clathrin reduced JEV infection, however, knockdown of caveolin-1 showed only a small effect on JEV infection and JEV entry was not affected by genistein. These results indicate that JEV endocytosis in PK15 cells is dependent on cholesterol and clathrin but not on caveolae.

## Results

### JEV infection is inhibited by the depletion of cholesterol

**Figure 1:**
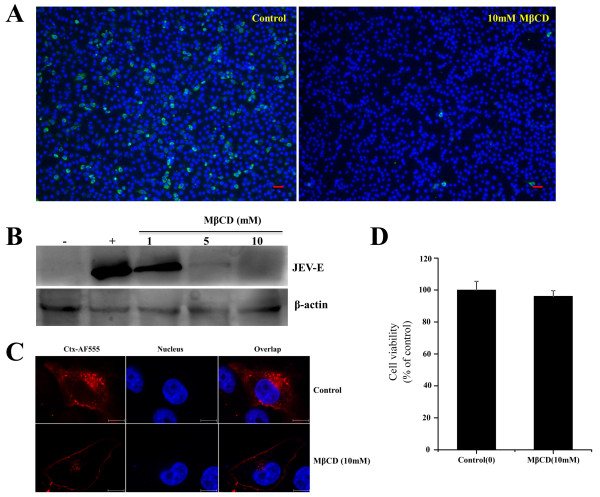
**Depletion of cholesterol by MβCD inhibits JEV infection. (A)** PK15 cells were treated with 10 mM MβCD for 1 h and infected with JEV in the presence of inhibitor; 36 hours post-infection (hpi), the cells were fixed and stained with anti-JEV E primary and Alexa488 anti-mouse IgG secondary antibodies. The nuclei were stained with DAPI. Scale bars, 50 μm. **(B)** The cells were treated with MβCD as described above and then infected with JEV; 36 hpi, the cells were lysed and processed for western blot analysis of JEV E protein. β-actin was used as an internal loading control. +, control cells untreated with MβCD; -, control without JEV infection. **(C)** PK15 cells were untreated (control) or treated with 10 mM MβCD for 1 h at 37°C and then incubated with 10 μg/ml AF 555-labelled Cholera toxin B (CTxB) for 30 min at 37°C. Nuclei were stained with DAPI. Bar, 10 μm. **(D)** PK15 cells were plated on 96-well plates and then either left untreated (as a control) or treated with MβCD (10 mM) for 6 h at 37°C. After treatment, cell viability was determined with a CCK-8 kit. The results are presented as the mean ± SD of three independent experiments.

Many viruses commonly use lipid rafts to enter host cells. Cholesterol is a prominent component of lipid rafts. Membrane cholesterol can be disrupted by pharmacological agents, in which MβCD extracts membrane cholesterol selectively [22], resulting in lipid raft disruption. Previous studies showed that the depletion of cholesterol could inhibit JEV infection during early stages [11,14,23]. To determine whether the removal of cholesterol affected the infection of PK15 cells with JEV, cells were treated with 10 nM MβCD and then incubated with JEV. After treatment, the internalization of JEV into cells was determined by immunofluorescence staining. As shown in Figure 1A, the treatment of the cells with 10 mM MβCD significantly decreased JEV infection compared with the untreated control (0 mM). In addition, after the cells were treated with 1, 5, or 10 mM MβCD and then infected with JEV, western blot analysis showed that the expression of JEV E protein was also inhibited. As shown in Figure 1B, JEV E protein expression was significantly inhibited by MβCD at the concentrations of 5 mM or higher. These concentrations of MβCD were previously reported to significantly deplete membrane cholesterol [11,24]. Cholera toxin B (CTxB) is internalized in a lipid-raft-dependent manner after binding to its receptor GM1 ganglioside [25]. To confirm that MβCD treatment resulted in inhibition of CTxB, we analyzed the effect of the drug on CTxB uptake. As shown in Figure 1C, untreated cells allowed CTxB uptake and showed a clear dotted perinuclear cytoplasmic fluorescence, whereas CTxB internalization was blocked when cells were treated with MβCD, confirming that lipid-raft-dependent endocytosis was inhibited. Cell proliferation and toxicological tests were determined by the CCK-8 kit. The results showed that the cells treated with 10 mM MβCD had no significant difference of absorbance from the control (Figure 1D). These observations suggested that JEV infection is dependent on the cellular membrane cholesterol content.

### Entry of JEV into PK15 cells is caveolae independent

**Figure 2:**
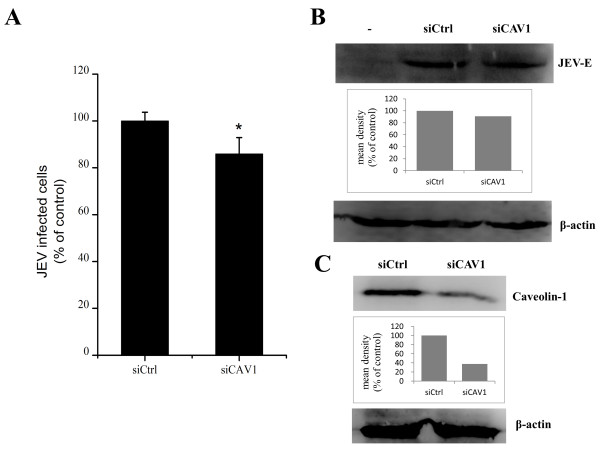
**Caveolin-1 is not required for JEV entry.** PK15 cells were transfected with siRNA targeting caveolin-1 or non-targeting siRNA (siCtrl) and infected with JEV at 48 h post-transfection, 36 hpi. **(A)** The percentage of internalized viruses of transfected cells was determined by flow cytometry and normalized to the value for the siCtrl. The results are presented as the mean ± SD of three independent experiments. **(B)** JEV E protein was analyzed by western blot analysis. β-actin was used as an internal loading control. -, control without JEV infection. The mean densities of protein bands were measured by ImageJ software, and the results for each gel are shown as a bar graph under the immunoblot. **(C)** Caveolin-1 knockdown cells were analyzed at 48 h post-transfection by the immunoblotting. The mean densities of the protein bands are shown as a bar graph under the immunoblot. *, *p*<0.05.

**Figure 3:**
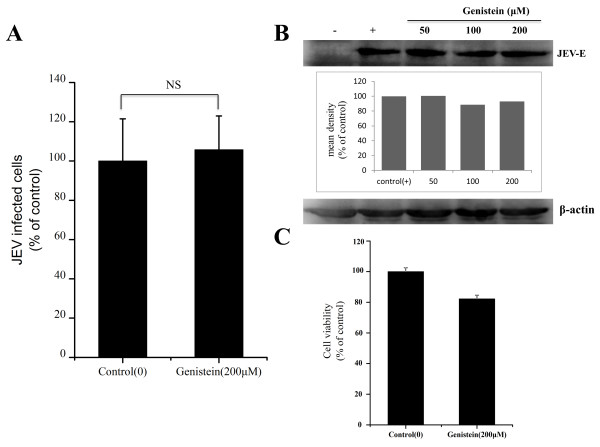
**Effects of genistein on JEV infection. (A)** PK15 cells were treated with 200 μM genistein for 1 h and infected with JEV in the presence of inhibitor; 36 hours post-infection (hpi), the cells were fixed and stained with anti-JEV E primary and Alexa488 anti-mouse IgG secondary antibodies. The percentage of internalized viruses of drug-treated cells was determined by flow cytometry and normalized to the value for the control. NS, not significant. **(B)** The cells were treated with increasing concentrations of genistein as indicated, then infected with JEV; 36 hpi, the cells were lysed and processed for western blot analysis of JEV E protein. β-actin was used as an internal loading control. +, control cells untreated with genistein; -, control without JEV infection. The mean densities of the protein bands are shown as a bar graph under the immunoblot. **(C)** PK15 cells were untreated (control) or treated with genistein (200 μM) for 6 h at 37°C. Cell viability was determined by CCK-8 kit. The results are presented as the mean ± SD.

Recently, it has been shown that JEV enters rat neuroblastoma cells through a caveolae-mediated endocytosis pathway [11]. caveolin-1 is the principal component of caveolae. The knockdown of caveolin-1 by RNA interference (RNAi) decreased the number of membrane caveolae [26]. To test whether the entry of JEV into PK15 cells occurs via caveolae-mediated endocytosis, PK15 cells were transfected with siRNA targeting caveolin-1 or control siRNA and then infected with JEV. A small (although statistically significant) effect on JEV infection was observed between the caveolin-1 knockdown cells and cells expressing control siRNA (Figure 2A). Western blot results showed that the cells transfected with siCAV1 did not have a marked inhibition in JEV E protein expression (Figure 2B). The silencing efficiency of siCAV1 were analyzed at 48 h post-transfection by western blot (Figure 2C). These results suggested a possible role for caveolae in JEV infection. However, genistein, a tyrosine kinase inhibitor known to disrupt caveola related endocytosis [27], had no effect on JEV infection at concentrations of 50, 100 and 200 μM (Figure 3A and B).

Cell viability tests were determined by the CCK-8 kit, minimal cellular cytotoxicity was observed in cells treated by 200 μM genistein compared with control (Figure 3C). These concentrations of genistein were previously reported to significantly inhibit JEV infection in rat neuroblastoma B104 cells [11]. These results suggest that, although caveolae-dependent processes may play some role in JEV infection, caveolae-mediated endocytosis is not the major route by which JEV enters the cell.

### JEV infects PK15 cells by clathrin-mediated endocytosis

**Figure 4:**
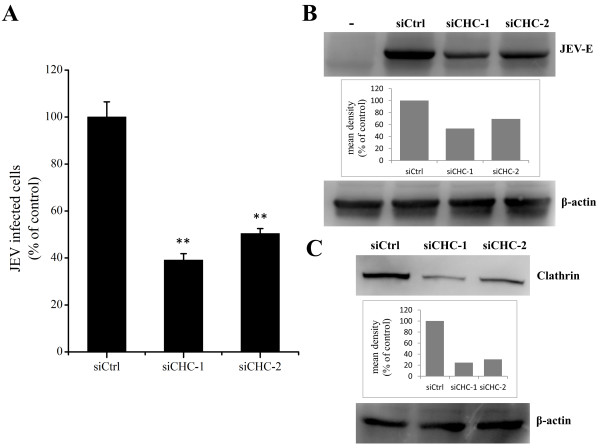
**JEV entry depends on clathrin.** PK15 cells were transfected with siRNA targeting clathrin heavy-chain (siCHC) or non-targeting siRNA (siCtrl) and infected with JEV at 48 h post-transfection. 36 hpi. **(A)** The percentage of internalized viruses of transfected cells was determined by flow cytometry and normalized to the value for the siCtrl. The results are presented as the mean ± SD. **(B)** JEV E protein was analyzed by western blot analysis. β-actin was used as an internal loading control. -, control without JEV infection. The mean densities of the protein bands are shown as a bar graph under the immunoblot. **(C)** Clathrin knockdown cells were analyzed at 48 h post-transfection by the immunoblotting for clathrin. The mean densities of the protein bands are shown as a bar graph under the immunoblot. **, *p*<0.01.

Early studies found that JEV entry into cells occurs by clathrin-mediated endocytosis and could be inhibited by chlorpromazine [13,14]. Thus, we examined whether clathrin-mediated endocytosis plays any role in JEV internalization. To specifically inhibit clathrin-mediated endocytosis, siRNAs were used to knockdown the expression of clathrin heavy chain (CHC). PK15 cells were transfected with two independent specific siRNAs against CHC or a control siRNA and then infected with JEV. The CHC knockdown cells showed a significant reduction in JEV infection compared with cells transfected with control siRNA (Figure 4A). Western blot analysis showed that the expression of JEV E protein was also decreased in cells transfected with siCHC compared with control cells (Figure 4B). The silencing efficiency of siCHC-1 and siCHC-2 were analyzed at 48 h post-transfection by western blot (Figure 4C). These experiments suggest that JEV internalization in PK15 cells can occur via a clathrin-dependent mechanism.

## Discussion

Lipid rafts are important for a number of cellular processes and play vital roles in virus entry [28]. Cholesterol is a prominent component of lipid rafts. Lipid rafts could be disrupted by depletion of cholesterol using chemical drugs, such as MβCD, nystatin and filipin [29-31], and some evidence suggests that cholesterol is involved in virus entry [24,32-34]. The inhibition of viral infection could be rescued by the addition of exogenous cholesterol. However, excess amounts of cholesterol block flaviviral infection [35]. Previous studies found that JEV could enter various cell types through a cholesterol-dependent pathway [11,13,14,23]. In the present work, PK15 cells were used to investigate the role of membrane cholesterol in JEV entry. Similar results showed that membrane cholesterol was an absolute requirement for JEV infection. Cellular entry is initiated when flaviviral gpE binds to cell surface receptors [36], and JEV internalisation occurs in a lipid-raft dependent manner [14]. Therefore, the cellular receptors for JEV may located within the lipid raft domains.

Viruses enter host cells through several endocytic pathways. Recent studies have found that JEV enters rat neuroblastoma cells via caveolae-mediated endocytosis [11]. Membrane cholesterol is required for caveolae formation, and caveolae-mediated endocytosis could be blocked by the depletion of cholesterol using MβCD [37]. The present study shows that JEV entry is inhibited after cells were treated with MβCD; thus, we speculated that caveolae was involved in JEV internalization. Therefore, drug treatment and small interfering RNA technology were used to validate whether JEV entry into PK15 cells was caveolae-dependent. Although the flow cytometry results showed that the cells transfected with caveolin-1 siRNA inhibited JEV infection by 15% (statistically significant), suggesting that caveolae-mediated pathway may play some role in the JEV entry into PK15 cells, JEV infection was not affected by genistein. We conclude that JEV entry into PK15 cells was caveolae-independent. In addition, the receptor for JEV on PK15 cells is still uncharacterized. It is possible that a very small part of receptors was localized in caveolae, thus, the knockdown of caveolin-1 had a small effect on JEV infection.

Furthermore, MβCD, which destroys lipid raft structure by depleting cholesterol, also inhibits clathrin-coated pit budding [38]. Therefore, we next examined the possibility of a clathrin-mediated pathway as an initial step in JEV entry into PK15 cells. Classic clathrin-mediated endocytosis is commonly used in virus internalization, and JEV entry occurs via a classic clathrin-mediated pathway in neural stem cells and Vero cells [13,14]. In this study, JEV was also found to enter PK15 cells through the clathrin-dependent endocytic pathway. Based on published data and the results reported here, we assume that JEV is likely to infect different cell types by distinct pathways. The cellular receptors of JEV may be different in different cell types. However, a correlation between the cell receptors used and their entry pathway was unknown. Several potential cellular receptors of JEV were identified [12,39,40], but the genuine receptors of various cell types need to be identified to better understand the JEV pathogenic mechanism and to search for new therapeutic targets.

## Conclusion

In the present study, we examined JEV infection using a chemical inhibitor and siRNA to disrupt different endocytic pathways in PK15 cells. Our results demonstrated that JEV entry was mediated by cholesterol- and clathrin-dependent, though not caveolin-1 dependent, pathways in PK15 cells. The mechanism of JEV entry in porcine cells was identified, and this research represents a new beginning for the development of new therapeutic targets in pigs.

## Materials and methods

### Cells and virus

PK15 and baby hamster kidney (BHK-21) cell lines (obtained from the American Type Culture Collection, Manassas, VA) were cultured in Dulbecco’s Modified Eagle Medium (DMEM, Thermo Scientific HyClone, Beijing, China) supplemented with 10% fetal bovine serum and maintained in a humidified incubator at 37°C and 5% CO_2_. The JEV attenuated strain SA14-14-2 (GenBank accession: AF315119.1) was used in this study. Virus was propagated in BHK-21 cells. To generate virus stocks, BHK-21 cells were grown in monolayers of 75-cm^2^ flasks, and the cells were infected with JEV until 90% confluence was reached. The cells were harvested when the cytopathic effect was extensive (48 h). Virions were collected through three freeze-thaw cycles and centrifugation. Virus titers were determined by the 50% tissue culture infectious dose (TCID50) [41], and the virus suspensions at a multiplicity of infection (MOI) of 1 were utilized for all infection experiments.

### Antibodies and reagents

Mouse anti-JEV E antibody was used for immunofluorescence assays and western blot analysis. Polyclonal rabbit anti β-actin, caveolin-1 and clathrin heavy chain antibodies were purchased from Cell Signaling Technology, Inc. (3 Trash Lane, Danvers, MA 01923, US). The Cell Counting Kit 8 (CCK-8), DAPI and genistein were purchased from Beyotime Biotechnology, Inc. (Jiangsu, China). Alexa Fluor 488-labeled goat anti-mouse IgG and Alexa Fluor 555-conjugated Cholera toxin B (CTxB) were purchased from Molecular Probes (Eugene, OR). HRP-conjugated goat-anti-rabbit and goat-anti-mouse secondary antibodies were purchased from Proteintech Group, Inc (Wuhan, China). Methyl-β-cyclodextrin (MβCD) was purchased from Sigma-Aldrich (St. Louis, MO) and dissolved in water.

### Drug treatments and virus infection

PK15 cells were plated on 6-well plates and grown to approximately 80% confluence at 37°C in a CO_2_ incubator. The cells were washed three times with phosphate-buffered saline (PBS) and then pre-treated with MβCD or genistein for 1 h at 37°C at various concentrations. After treatment, the cells were infected with JEV for 1 h at 37°C in the presence of the chemical. The cells were washed to remove excess virus and drug, then further incubated in cell culture medium containing 2% FBS at 37°C with 5% CO_2_. Western blot and immunofluorescence staining analysis for JEV E protein expression were performed 36 h post-infection.

### RNA interference experiments

For small interfering RNA (siRNA) analysis, siRNA oligonucleotides with specificity for porcine CAV1 and clathrin heavy chain (CHC) and non-targeting siRNA (siCtrl) were synthesized from GenePharma (Shanghai, China). The target sequence of siCAV1 was as follow: siCAV1-1 5’- CACACAGUUUCGAUGGCAUCUTT-3’. Sequences of siCHC-1 (equal mixture of the two RNA duplex oligonucleotides) were as follows: 5’-AAGCUGGGAAAACUCUUCAGA-3’ , 5’-UAAUCCAAUUCGAAGACCAAU-3’ [42], siCHC-2: 5’-GGCCCAGGUGGUAAUUAUUUU-3’. PK15 cells were transfected with 50 nM siRNA using Lipofectamine 2000 (Invitrogen) according to the manufacturer’s instructions. Subsequent experiments were performed 48 h post-transfection. Knockdown efficiencies were quantified by western blot analysis.

### Western blot analysis

Cells were washed with PBS three times and lysed in a modified radioimmunoprecipitation assay (RIPA) lysis buffer (50 mM Tris (pH 7.4), 150 mM NaCl, 1% Triton X-100, 1% sodium deoxycholate, 0.1% SDS, 1 mM phenylmethylsulfonyl fluoride [PMSF]). Protein concentrations were determined with a BCA Protein Assay kit (Solarbio, China). An equal amount of protein lysate was separated by 12% SDS-polyacrylamide gels and transferred to PVDF membranes (Millipore, Bedford, MA). The membranes were blocked with 5% nonfat milk in tris-buffered saline containing 0.1% tween-20 (TBST) and then incubated with primary antibodies overnight at 4°C. The membranes were washed three times with TBST (each wash was 10 min), and HRP-conjugated goat anti-rabbit or goat-anti-mouse secondary antibody (1:3000 dilutions in blocking buffer) was added. Bound antibodies were visualized by SuperSignal West Pico chemiluminescent substrate (Pierce, Rockford, IL). The mean densities of protein bands were measured by ImageJ software (National Institutes of Health, Bethesda, Maryland).

### Immunofluorescence assays

The cells were fixed with 4% paraformaldehyde for 20 min at room temperature (RT) and permeabilized with 0.2% Triton X-100 for 15 min. The cells were then incubated with a blocking buffer (PBS containing 5% bovine serum albumin [BSA]) at 37°C for 30 min. After three washes with PBS, the cells were stained with anti-JEV E mouse antibody at room temperature for 1 h. After being washed with PBS, the cells were incubated with Alexa Fluor 488 conjugated goat anti-mouse antibody (1:200 dilutions in blocking buffer) IgG. The nuclei were stained with DAPI.

### Flow cytometry

PK15 cells infected with JEV were washed one time with PBS, detached and transferred to 1.5 ml centrifuge tubes. The cells were centrifuged at 1000 rpm/min for 10 min and fixed with 4% paraformaldehyde for 15 min at room temperature. After being permeabilized with Triton X-100, the cells were incubated with anti-JEV E mouse antibody overnight at 4°C. The cells were washed 3 times with PBS then incubated with Alexa Fluor 488 goat anti-mouse IgG at 1:200 for 1 h at room temperature. The cells were washed with PBS, resuspended in 500 μl PBS and analyzed using FACScan flow cytometer with CellQuest pro software (BD Biosciences, San Jose, CA). The cells were counted as infected if their fluorescence densities were greater than the intensity of the uninfected cells. The amount of infected cells relative to the untreated or siCtrl-transfected controls was given as percent infection. At least 10,000 cells were analyzed per sample.

### Statistical analysis

The results were presented as the mean ± standard deviation (SD). Statistical significance was assessed by Student’s t-test, and statistical significance was ascribed when *p*<0.05.

## Competing interests

The authors declare that they have no competing interests.

## Authors’ contributions

SBY carried out most of the experiments and drafted the manuscript. MHH, XYL and XDL participated in part of experiments. BF and SHZ designed the study, supervised the work and edited the final version of this manuscript. All authors have read and approved the final version of the manuscript.
